# Abstractive text summarization of low-resourced languages using deep learning

**DOI:** 10.7717/peerj-cs.1176

**Published:** 2023-01-13

**Authors:** Nida Shafiq, Isma Hamid, Muhammad Asif, Qamar Nawaz, Hanan Aljuaid, Hamid Ali

**Affiliations:** 1 Department of Computer Science, National Textile University, Faisalabad, Pakistan; 2 Department of Computer Science, University of Agriculture Faisalabad, Faisalabad, Pakistan; 3 Computer Sciences Department, College of Computer and Information Sciences, Princess Nourah bint Abdulrahman University, Riyadh, Saudi Arabia

**Keywords:** Urdu, Abstractive summarization, LSTM, BERT2BERT, Pars-BERT, Seq-to-Seq

## Abstract

**Background:**

Humans must be able to cope with the huge amounts of information produced by the information technology revolution. As a result, automatic text summarization is being employed in a range of industries to assist individuals in identifying the most important information. For text summarization, two approaches are mainly considered: text summarization by the extractive and abstractive methods. The extractive summarisation approach selects chunks of sentences like source documents, while the abstractive approach can generate a summary based on mined keywords. For low-resourced languages, *e.g.*, Urdu, extractive summarization uses various models and algorithms. However, the study of abstractive summarization in Urdu is still a challenging task. Because there are so many literary works in Urdu, producing abstractive summaries demands extensive research.

**Methodology:**

This article proposed a deep learning model for the Urdu language by using the Urdu 1 Million news dataset and compared its performance with the two widely used methods based on machine learning, such as support vector machine (SVM) and logistic regression (LR). The results show that the suggested deep learning model performs better than the other two approaches. The summaries produced by extractive summaries are processed using the encoder-decoder paradigm to create an abstractive summary.

**Results:**

With the help of Urdu language specialists, the system-generated summaries were validated, showing the proposed model’s improvement and accuracy.

## Introduction

In natural language processing (NLP), the summarization of text is a difficult job. It aims to do more manageable reading and search information from many papers by creating smaller versions without losing significance. Because of the Internet’s fast expansion over the past two decades, data availability news, articles, and book reviews can all be found on the Internet (Burney, Sami & Mahmood, 2012), which will increase rapidly. There is a significant increase in textual data and it is continuously multiplying due to the overwhelming volume of data. Users use search queries to find information on the Internet. Even still, the user must visit numerous web pages, which takes time and is like a headache to find the information they require. So, to avoid this headache, deal with this massive amount of data, and get the information (Kumar & Rani, 2021) from an entire article in the shortest way, a method is introduced, termed text summarization. Based on the type of summary that is produced, text summarization can be classified into two categories: abstractive and extractive text summarizing. To excerpt major portions of the source text verbatim, extractive summarization mostly relies on statistical or linguistic factors (Suleiman & Awajan, 2020). While the abstractive summarization restates the obtained text to produce words that are not certainly included in the source text, as opposed to duplicating some sections of the original text (Liang, Du & Li, 2020). Generating the summary using natural language processing and advanced machine learning algorithms makes abstractive text summarizing more difficult than extractive text summarization. The materials must be interpreted and semantically evaluated to provide an abstractive summary (Azmi & Altmami, 2018).

Certain systems also employ convolutional neural networks to examine semantic characteristics (Wang et al., 2020). However, because the abstractive-generated summary closely resembles the human-derived summary, abstractive summarizing is preferable to extractive summarization. The summary is hence more insightful (Sunitha, Jaya & Ganesh, 2016). No matter the method of summary, both types of summaries require that these have certain traits. The following are the areas’ primary traits: even if the material is lengthy, the produced summary and the original text’s sentence structure and meaning must coincide (Muhammad et al., 2018). A compressed text summary may be produced using the two levels of encoder and decoder found in the sequence-to-sequence paradigm. Additionally, the produced summary should convey the original text’s whole sense. While maintaining the same meaning, the summary’s size must be shorter than the original text (Burney, Sami & Mahmood, 2012; Liang, Du & Li, 2020). Finally, it is important to reduce the amount of repetition in the summary that is created.

According to the study, this model is based on a deep neural network. This may extract keywords associated with a topic, which are then utilized as input. Modern breakthroughs in deep learning have recently been made in NLP applications. Because feature space is sparse with high dimensions, the machine learning algorithms such as support vector machines and logistic regression were employed for handling NLP complications narrowly (Young et al., 2018). Deep learning approaches have recently been extensively cast-off in abstractive text summarization due to their promising outcomes.

The proposed method in this study is based mostly on the seq2seq recurrent neural network (RNN) architecture. Seq2seq mapping is used in NLP tenders like text summarization and machine transformation to plot two arrangements of fonts, words (Fischer, 2004), and expressions in a neural network. To perform this experiment, a dataset consisting of more than 1 million news stories and their summaries is considered. It is the largest dataset available for performing NLP experiments in the Urdu language. The text is the initial sequence in text summarization, and the summary is the second sequence. Deep learning techniques are used to address the issue of high-level dimensionality and the sparseness of the characters. An RNN, on the other side, is made up of a series of hidden states, each with its output that is fed into the next stage (Widyassari others, 2022). The sequential aspect of an RNN makes it easier to analyze data sequentially, like identifying the connotation of a term in a sentence depending on the preceding or following words. The productivity of all previous secreted states is accumulated in the last hidden state of an RNN to form the context vector (Bhaduri, 1990). The vector depiction of each expression in the manuscript is mixed with the productivity of the concealed state before it at each hidden stage of the encoder. The word implanting of the “SOS” sign is the word implanting, and the resulting summary’s first word is the output. The framework vector is the input of the initial unknown state in the decoder. Numerous word embedding mockups, such as word2Vec and GloVe, have recently been used. Extractive summarization models don’t understand sentence meaning (Dwi Sanyoto, 2017). The summary is created by concatenating keywords, phrases, and sentences.

Our proposed abstractive text summarization methodology is alienated into three stages: in the first phase, the dataset is collected, and preprocessing is done. In the second stage, extractive text summarization is done; in the third stage, abstractive text summarization is done. For abstractive summarization, the encoder–decoder model is considered. Three layers of the encoder and a single layer in the decoder make up the suggested model. The encoder–decoder utilizes long short-term memory (LSTM). The following are the inputs to the word embedding of encoder layers: the initial layer’s input text, the input text’s keywords in the next layer, and the input text’s name entities in the final layer. On the other hand, the word vectors generated using word embedding serve as the input for the decoder layer. A summary is created by the decoder s using the global attention method.

The remaining article is structured as follows. Related work is described in the “Related work” section. While in the “Problem statement and motivation” section, the problem statement and motivation are provided. In the next section, the research contribution is addressed. The “Suggested model” section presents the suggested model. The evaluation and outcomes of the experiment are described next. The “Conclusions” section presents the conclusion.

### Related work

**Figure 1:**
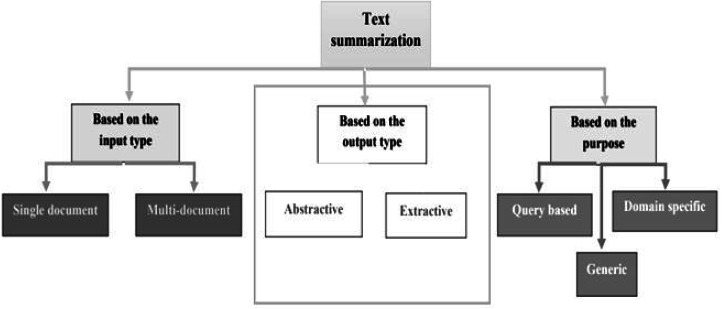
Text summarization approaches.

**Figure 2:**
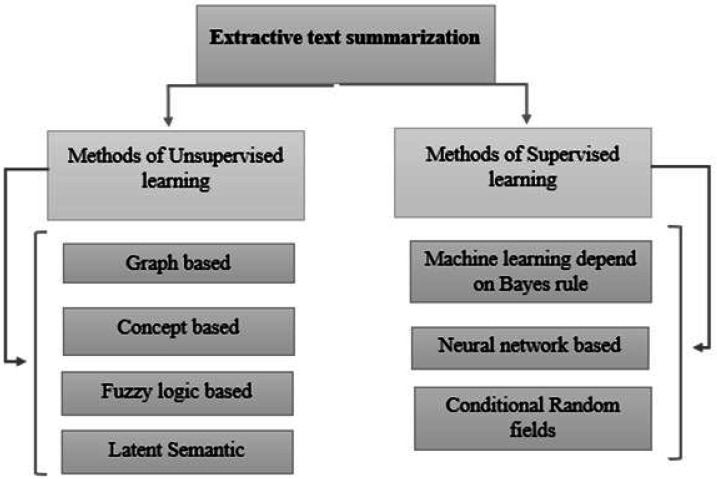
Overview of extractive text summarization.

**Figure 3:**
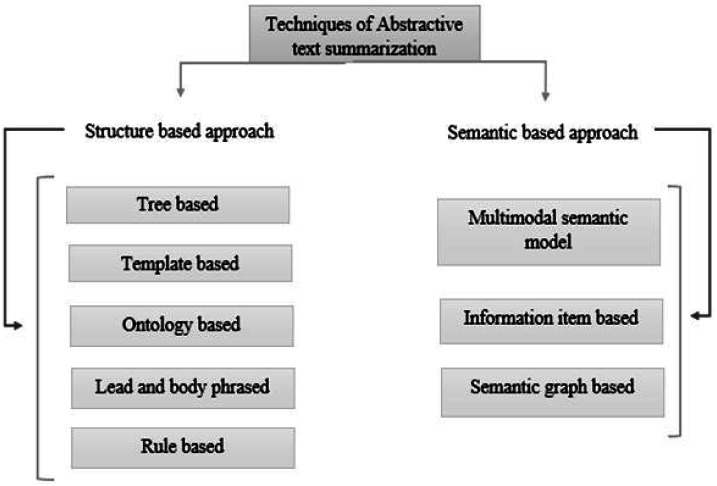
Types of abstractive text summarization.

In recent years, Urdu linguistics has achieved significant progress. A substantial volume of data is generated by numerous portals and news websites day after day. Without knowing the meaning of the phrases, extractive summarization methods construct summaries (Dwi Sanyoto, 2017). As a result, abstractive summaries are more precise than extractive summaries (Kiyani & Tas, 2017). However, because statistical approaches are faster than linguistics procedures, the extracted summary is generated faster. Abstractive and extractive approaches for patent labelling have been examined (Moratanch & Chitrakala, 2016). Overall, comparing abstractive *versus* extractive (Dalal & Malik, 2013) approaches is difficult for various reasons. The approaches to text summarization are shown in Fig. 1. These are divided into extractive and abstractive text summarization based on the output type. The overview of extractive summarization types is depicted in Fig. 2 and the types of the overview of abstractive summarization are shown in Fig. 3.

**Unsupervised learning**: These methods do not require human summaries (user input) to determine the crucial aspects of the content.
  **Graph-based approach:** Since graphs may effectively reflect the document structure (Iyer, Chanussot & Bertozzi, 2018), these models are frequently employed in document summarization.
  **Concept-based approach**: This approach extracts (Hashemi, Tyler & Antonelli, 2014) theories from texts using external knowledge bases like HowNet and Wikipedia.
  **Fuzzy logic-based approach**: Sentence length, sentence similarity (Ropero et al., 2012), and other textual properties are inputs for the fuzzy logic technique that are later provided to the fuzzy system.
  **Latent semantic analysis**: The technique known as Latent Semantic Analysis (LSA) (Ozsoy, Alpaslan & Cicekli, 2011) allows text summarizing tasks to extract latent semantic constructions of sentences and phrases.
**Supervised learning:** At the sentence level, techniques linked to supervised extractive summarization are based on a classification strategy (Wikipedia, 2022). The model is taught by using examples to distinguish between non-summary and summary phrases.
  **Machine learning depends on the Bayes rule:** The machine learning method sees text summarization as a classification problem (Brownlee, 2019). The sentences are limited to non-summary or summary based on each attribute.
  **Neural network based:** It considers a RankNet-trained neural network with a two-layer and backpropagation approach (Kamper et al., 2015). To score the sentences in the document, the neural network system must first perform feature extraction on sentences in the test and training sets. This is done in the first phase, which uses a machine-learning approach for labelling the training data.
  **Conditional random fields:** A statistical modelling strategy called conditional random fields (Macherla, 2020) focuses on using machine learning to produce structured predictions.

**Structure-based approach**: It utilizes deep learning algorithms to choose the crucial passages from the original documents (Garg & Saini, 2019).
  **Summarization based on tree method:** (Kikuchi et al., 2014) uses a dependency tree to describe the text and information from the source text.
  **Summarization based on template method:** It is a method that gives the end user the freedom to design a template for the information that should be in summary (Oya et al., 2014). The template includes POS markers like adverbs, verbs, and nouns, among others, and the end user may define the method by which the sentences should appear in summary.
  **Ontology-based method:** The method for developing ontologies (Jishma Mohan et al., 2016) uses data preprocessing, semantic information extraction, and ontology development**.**
  **Lead and body phrased method:** It depends on the “insert and replace” process, which uses core sentences to replace the leading phrase and comparable syntactic head chunks at the beginning of each step (Sciforce, 2019).
  **Rule-based method**: Using this method (Vodolazova & Lloret, 2019), the textual materials are condensed by being shown as a collection of specifics.
**Semantic-based approach:** (Shahzad et al., 2022) In the semantic-based approach, ideas relevant to phenotypes are taken from the domain knowledge base’s class hierarchy and a semantic similarity metric determines their significance.
  **Multimodal semantic model:** In this method (Chen & Zhuge, 2018), the subject (images and manuscript data) of one or more documents is represented by a semantic unit that extracts the subject content and correlations among the topics.
  **Information item based**: Using the original text’s sentences as a starting point, Using this method, the original text’s abstract representation is used to construct the data for the summary.
  **Semantic graph based:** The Rich Semantic Graph (RSG) builds a semantic graph on the source content, condenses the semantic network, and then provides an exhaustive abstractive summary from the condensed semantic graph.

### Problem statement and motivation

Considering advancements in software and hardware technologies and the expanded use of machine learning models, text summarization has changed over the past ten years as an NLP application (Yao et al., 2020). Extractive and abstractive summarization are the two basic technical subcategories of text summarization. Despite the vast quantity of information in Urdu web papers, there are many issues with text summaries in the Urdu language. To increase the readability of the Urdu language, it needs to generate a summary that retains the original text’s meaning. For Urdu languages, only extractive summarization is done by various algorithms and models, but not abstractive summarization.

Since abstractive summarizing of Urdu text is more difficult than extractive summarization, this paper’s primary contribution is the suggestion of an abstractive paradigm for summarizing Urdu texts. And improve the results for Persian language abstractive summarization. Due to the resulting summary’s abstractive character and Urdu’s intricate morphology, creating such a model is challenging. The suggested model was confronted with two primary obstacles: the first obstacle was the dispersed interpretation of the writing that considered the complexity of the Urdu language. The second problem was finding appropriate assessment metrics to judge the result of the generated summary.

## Research contribution

In 2015, the first use of deep learning methods to abstract English text summarization was proposed (Dwi Sanyoto, 2017). The best that we can tell, however, abstractive Urdu text summarization still does not employ deep learning. The overall goal of this research is

 •    To generate a meaningful and concise summary that includes new words and sentences for Urdu languages. Which enhances the readability and grasp of the overall meaning of the source document by abstractive text summarization.

 •  To improve the correctness and readability of generated summaries for the Persian language. This work has focused on utilizing the abstractive text summarization model. It considers the source data or other documents for summary generation. Two summaries are generated. The first summary is generated by the philologist, while the model generates the other summary. The model-generated summary was compared with the summary generated by the philologist. The generated summary can be of multiple documents or a single document.

## Proposed Research Methodology

**Figure 4:**
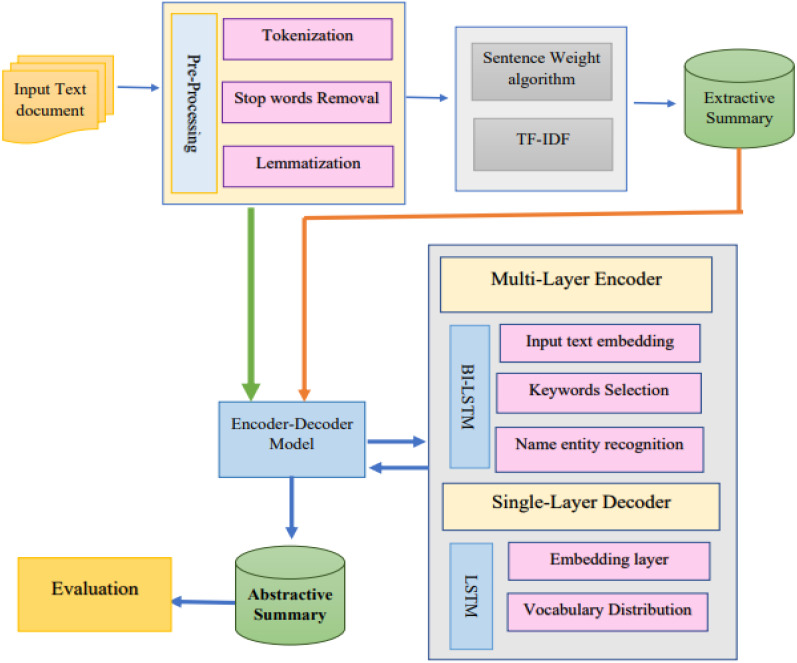
Proposed methodology framework for abstractive Urdu text summarization.

The suggested architecture’s structure is divided up into many parts. The text is first pre-handled using common NLP techniques, including “normalization, tokenization, lemmatization, POS tagging, and stop-word elimination”. After the preprocessing, the characteristics are retrieved, and sentences are sorted according to their load and the frequency with which each phrase or token occurs. The encoder–decoder receives the final summary and creates an abstract summary. The framework of the proposed methodology is presented in Fig. 4.

### Dataset

To conduct this research 1 Million news dataset is used, categorized into four types: Sports, Science & Technology, Business & Economics and Entertainment. It consists of long news text stories and their short summaries. The dataset incorporates news to date, URL, web source and the number of characters in news stories. We have used 70% of the data for training the model and the rest of the 30% for testing. It is the largest dataset in the Urdu language for performing natural language processing tasks.

### Pre-processing

**Figure 5:**
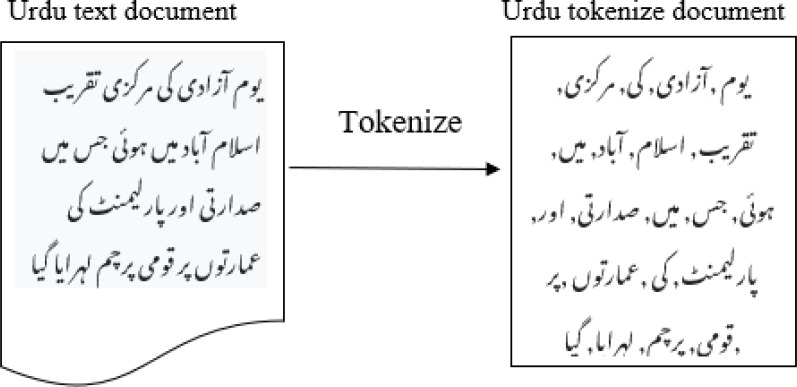
Tokenization of Urdu sentence.

Typically, text preprocessing comes first in any NLP task. For the English language, a variety of open-source programs are available. It is still very difficult when it comes to Urdu or any other related languages, including Persian, Arabic, and Pashto. However several e-libraries are available for the Urdu language, but due to their poor accuracy, preprocessing still requires a lot of work. Normalizing textual texts is an important step in preprocessing. For instance, several nouns in the Urdu language contain digraphs, such as (( 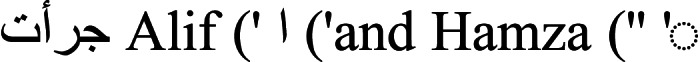 Alif (’ (’and Hamza (” ’ )) which, while being separate alphabets, have been employed as a separate notes. Separating these two letters is necessary for further processing. With relation to the syntactic organization, Urdu is a rich language. Several words may be written with or without a space. It is ensured that there are suitable gaps between words and pronunciations; The content normalization module will also remove diacritics and accents. It is converting a phrase into different forms, such as a list of tuples or a list of words, each of which has a form (word, tag). One of the crucial steps in-text pre-treating is tokenization. Tokenization can be considered as breaking down a text into specific or different terms, whether it sentences, phrases, paragraphs, or the complete document, as shown in Fig. 5. Tokenization assists in interpreting the text’s meaning by looking at the word order.

Tokenization involves breaking up long statements into sentences and words in two fundamental phases. By looking for word and sentence endings, tokenization of phrases is produced. These word counts are used as the start and stop positions for words. Exclamation points (!), question marks (?), and full stops (./-) are used as delimiters to separate paragraphs. Further processing, like lemmatization, is carried out based on these tokens. Lemmatization aims to break down words into their most fundamental components. As an illustration, the word ” ” 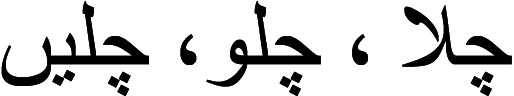 ” is ” 
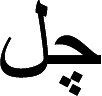
 ” has the origin ”.” During the lemmatization procedure, the prefixes and suffixes are shortened, leaving only the word’s stem, As depicted in Table 1. Another crucial stage in text preparation is lemmatization. It is important to understand the context in which the word is used.

Stop words are defined as having significance in the context of semantic values. Removing these terms makes our text more focused on the key information by eliminating the low-level information. Only grammatically constrained orders to utilize these words. Stop words frequently appear in documents and their occurrence in phrases has little semantic significance. These words encompass a significant collection of archives without any meaningful value. The stop words, thus, for better language description, should be removed.

Urdu common stop words are 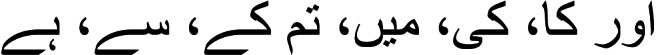 . The content words (tokens) left after the stop-words are eliminated and then are available for processing as shown in Table 2.

### Extractive summaries

After the pre-initialization operation is finished, the text’s characteristics are extracted. Extractive summarization is the process through which key phrases founded on a benchmark are chosen to give a comprehensive summary to communicate the original text’s key concept accurately.

**Table 1:** Some extracted suffix.

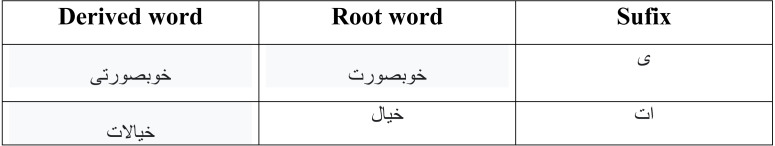

**Table 2:** Sentences with and without stop words.

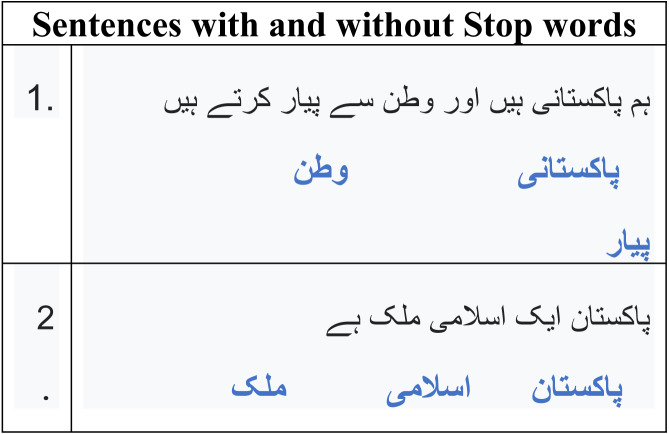

#### Sentence weight algorithm

For feature extraction, the text is prepared after preprocessing. To provide an extractive summary for capturing the core concept of the innovative text, extractive summarization selects pertinent phrases based on several feature sets. Considering extractive summarizing, the weights are assigned to the source phrases, and the heavily weighted sentences are assigned a higher rank for a summary generation. Sentence weight algorithms are used for important sentence retrieval, a statistical method depending on the weights given to each phrase. Sentences are rated based on the ratio of content words to total words. According to Eq. (1), Let W = {L_1_, L_2_, L_3_,……L_N_} represents the supplied Urdu source text, where n represents the total number of sentences, and Li represents a single phrase. Tokens are created for each phrase. As previously said, Li is designated as /Important words are chosen by removing stop words, /indicated as contains all the previously absent words. The ratio of all filtered words to all words is how the weight of Li, represented as w, is calculated.

(1) \begin{eqnarray*}{L}_{i}^{w}= \frac{ \left\vert {I}^{i} \right\vert }{ \left\vert {L}_{i} \right\vert } .\end{eqnarray*}

#### Word frequency algorithm

The statistical technique recognized as Term Frequency Inverse Document Frequency (TF-IDF) similarly explains the importance of a term in a document. The incidence with which a word seems in the provided text consistently increases the weight of the TF-IDF. However, it is assessed by the event’s token that appears in the text and helps identify which terms are more common than others. Consider w = {word_1_, word_2_, ……word_n_}

Which is the total number of words and *W*_*n*_ is the overall sum of document words. As shown in Eq. (2).

(2) \begin{eqnarray*}TF= \frac{W}{{W}_{n}} .\end{eqnarray*}

For the calculation of IDF, compute the entire number of credentials D_n_ by the document frequency D_f,_ As shown in Eqs. (3) and (4).

(3) \begin{eqnarray*}IDF=\log \nolimits ( \frac{{D}_{n}}{{D}_{f}} )\end{eqnarray*}

(4) \begin{eqnarray*}TF-IDF=TF\times IDF.\end{eqnarray*}

The assessment of each token’s TF-IDF value determines whether a phrase is urgent. The sentences are then ordered from lower to higher TF-IDF values. Focus is placed on selecting the sentences with a high TF-IDF value for a comprehensive summary.

### Abstractive summary

For most NLP applications that use data sequences, such as machine translation and text summarization, the Seq2Seq recurrent neural network (RNN) architecture recently rose to the top. The encoder–decoder paradigm is employed when one sequence serves as the contribution and another as the output.

**Figure 6:**
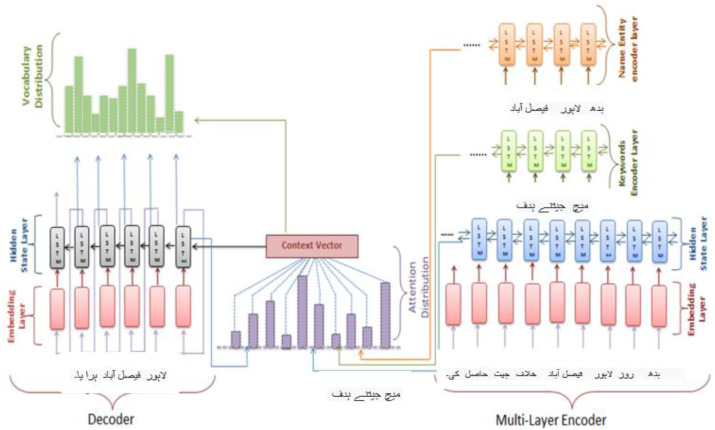
Multilayer encoder abstractive Urdu text summarization.

The vanishing gradient problem, which may be fixed by employing the LSTM, may affect the RNN. In this study, we’ll employ a model made up of an encoder–decoder LSTM. On the other hand, the proposed method utilised a multilayer encoder instead of a single-layer encoder. Three hidden state layers make up the multilayer encoder: the input text’s secreted conditions are on the top layer, the text’s secret states of its keywords are on the bottom layer, and the text’s hidden states of its name entities are on the top layer. The inputs for the three layers are word embedding of the text words, keywords, and name entities. For creating word vectors, 128 dimensions were considered as depicted in Fig. 6. Bi-directional LSTM units make up the three encoder layers’ hidden states. The input text order {tx = tx_1_, tx_2_, tx_3_, …, tx_n_} is generated by the first layer from right to left and is drawn to the hidden states {hs = hs_1_, hs_2_, …, hs_n_,} correspondingly. The hidden states {h_k_ = hk1, hk2, hk3, …, hkq} are formed in the second layer and correspond to the depiction of the documents {k = k1, k2, k3, …, kq}. The keywords representation kv is created by concatenating the last forward keywords’ hidden state and the last backward keywords’ hidden state. The text name entities {nne = ne1, ne2, ne3, …, nee} are present in the text and provided as input to the hidden states hne = hne1, hne2, hne3, …, nee are represented in the final layer. To create the name entity representation nev, the most recent forward hidden state and the most recent backward hidden state are concatenated. In contrast, the decoder’s hidden states are made up of a single layer of a unidirectional LSTM. The decoder gets the word embedding produced by the decoder for the preceding word.

For the Persian language (Liaqat & Hamid, 0000), our critical commitment is that we extensively experiment with many things with various settings to combine BERT, GPT, and RoBERTa pre-prepared stations before launching our model, which is based on the transformer.

**Figure 7:**
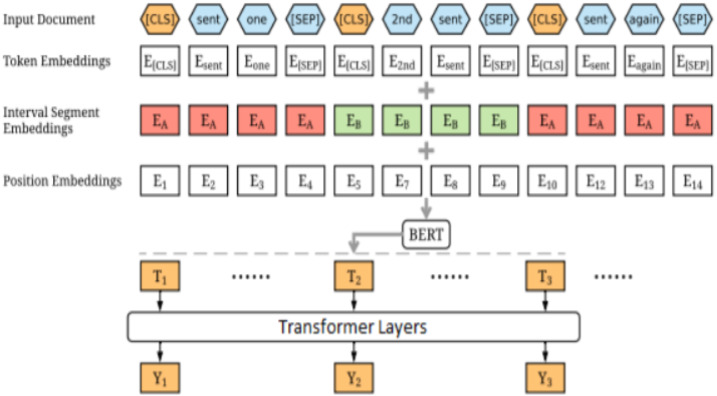
Bert Arch.

The models periodically report significant improvements on initial prototypes that use managed pre-planned models. More importantly, this simple process yields brand-new top-of-the-class outcomes in machine analysis, note synopses, phrase delivery, and sentence combining. The results of the suggested technique also demonstrate that a trained encoder is an essential component of arranging assignments. These tasks frequently profit from distributing the load across the encoder and the decoder. This study used more than 300 tests and several TPU v3 h in total, which increased the likelihood that these text-age-ready models’ language-displaying and cognition abilities would alter. This study ensures that NLP analysts and experts will gather useful information from the suggested outcomes as they take on the various seq2seq tasks. Using Word Piece, this research matched its content to the pre-prepared jargon of BERT, as seen in Fig. 7. The data collected from various resources is shown in Table 3 and the training time is illustrated in Table 4.

**Table 3:** Data collections from various sources.

#	Source	Total true sentences
1	Chetor	166,312
2	Ted Talks	46,833
3	Persian Wikipedia	1,878,008
4	Digikala	177,357
5	Eligasht	214,328
6	BigBang Page	3,017
7	Miras-Text	35,758,281
8	Books	25,335

**Table 4:** Training time.

Downstream task	Dataset	Train time (hh:mm:ss)
Text classification	Digikala Magazine	00:10:40
	Persian News	00:21:15
NER	PEYMA	00:45:19
	ARMAN	00:30:57
Sentiment Analysis	Digikala sentiment	1:00:25
	Snappfood sentiment	1:00:22
	DeepSentiPers Binary	00:08:00
	DeepSentiPers Multiclass	00:15:00

## Results and Discussions

**Figure 8:**
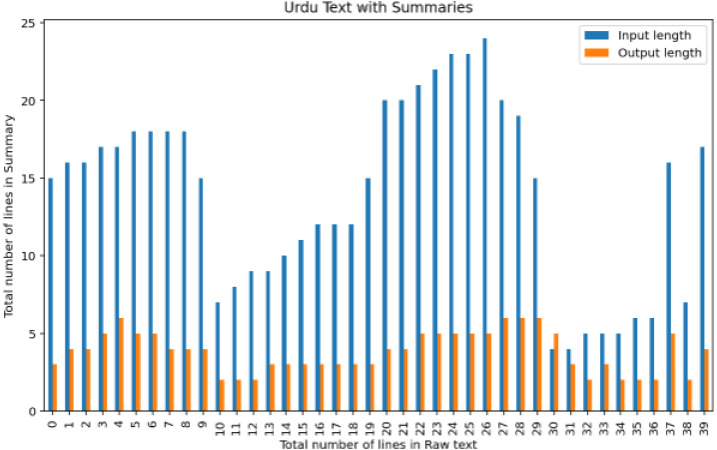
Analysis of the number of lines in raw text with a number of lines output summary.

For instance, the suggested summary extent ratio should be between 33 and 40 per cent, while some summaries have a size ratio as high as 80 per cent of the specified text. Roughly forty articles are divided into various areas, as shown in Fig. 8.

 For a computer to be completely well-matched with human language, its innate language abilities, and its terminology is always an enormous task.

**Figure 9:**
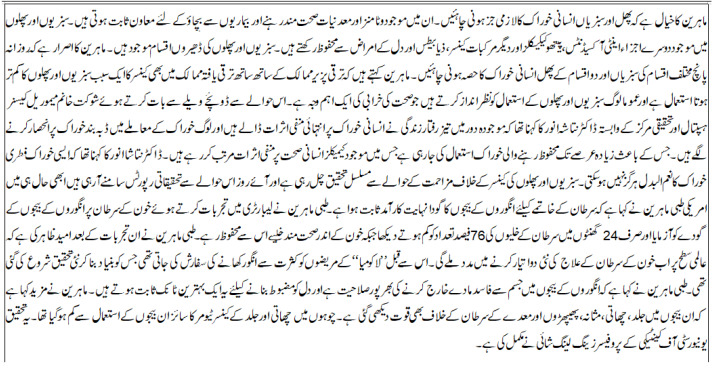
Input source test document.

**Figure 10:**
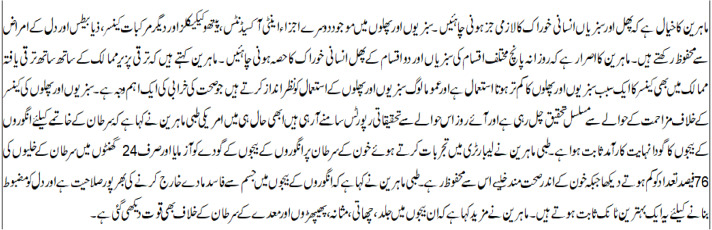
Extractive summary from the input source document.

There were 20–21 lines of Urdu text in the assessment document. The summary was created over a Sentence weight algorithm of 11 lines close to 50% of the definite document, as shown in Fig. 9. The most important sentences were grouped and categorized according to sentence weight and TF-IDF technique. As a result, the created summary kept the core ideas of the original text., which had 10–12 lines, or around 51% of the source document depicted in Fig. 10. The derived summary was noted to have a mix of meaningful and random sentences. The most laborious wordy sentences were picked.

**Figure 11:**
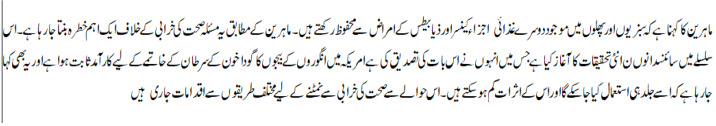
Abstractive summary from extractive summary.

The abstractive summary, which makes up one-half of the extractive summary, is then created using this summary. This is so that the Encoder-Decoder model can produce the abstractive summary by using Bi-LSTM on the encoder side and LSTM on the decoder side. The abstractive overview uses a variety of additional vocabulary to show how the suggested system may translate sentences written in Urdu. The resultant abstractive summary might be said to be brief and compact. This abstractive summary is a quarter of the source test document, as shown in Fig. 11.

The suggested model and its variants are trained and tested using the publically available dataset (Hussain et al., 2021). Quantitative measurements are also used to assess the models. In addition to the metrics suggested in this research, the ROUGE assessment measure is employed in the quantitative evaluation. Recall-Oriented Understudy for Gisting Evaluation is referred to as ROUGE. It primarily consists of a collection of measures for assessing Automatic Text Summarization. The findings indicate that models using dual and multilayer encoders perform better than single-layer encoders. This is since employing stacked LSTM, which consists of many levels of LSTM, enables the hidden states at each layer to operate on distinct timescales. The models built on stacked LSTMs were enhanced, notably in predicting sequence models like text summarization. Each layer improves the context vector’s quality by offering extra information. Additionally, in text summarization, every layer might include fresh characteristics connected to the input text. Finally, ROUGE1, ROUGE2, or ROUGE-L measures are used to assess the resulting summary. Table 5 displays the model’s accuracy for Rouge.

**Table 5:** Model’s accuracy for Rouge.

%	**Precision**	**Recall**	**F-Measure**
ROUGE 1	79	30	43
ROUGE 2	53	16	25
ROUGE L	41	15	23

**Figure 12:**
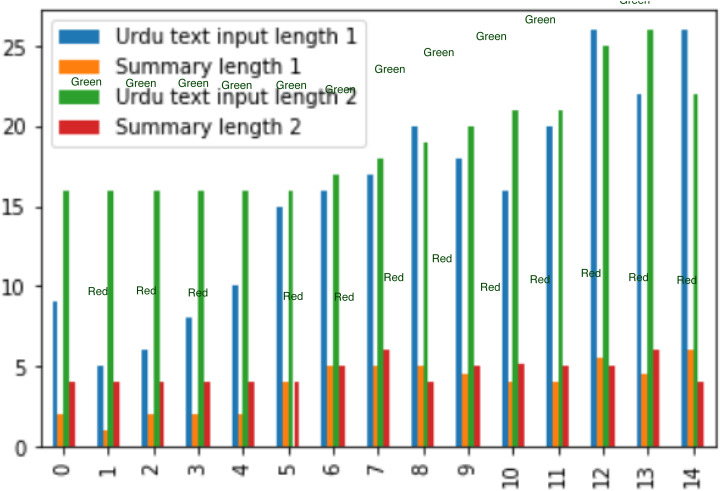
Comparison of dataset results.

For experiment two, datasets are taken, which are of different lengths, varying from 8 lines to 26 lines and its summary length is about its quarter length. The bar graph is created when comparing two Urdu datasets, as depicted in Fig. 12.

**Figure 13:**
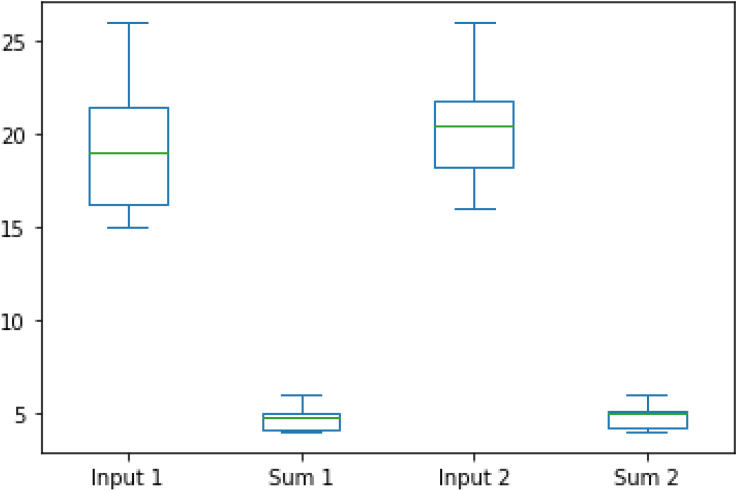
Boxplot of dataset comparison.

The boxplot of the dataset comparison is depicted in Fig. 13.

For the Persian language, consider the F1 score while evaluating the proposed models due to the uneven class dispersion. Let P show accuracy and R show memory, and then the weighted normal approach, which depends on the number of real marks in each class using the following conditions, determines the f1 score.

(5) \begin{eqnarray*}F1-Score=2\times \frac{P\times R}{P+R} .\end{eqnarray*}

**Figure 14:**
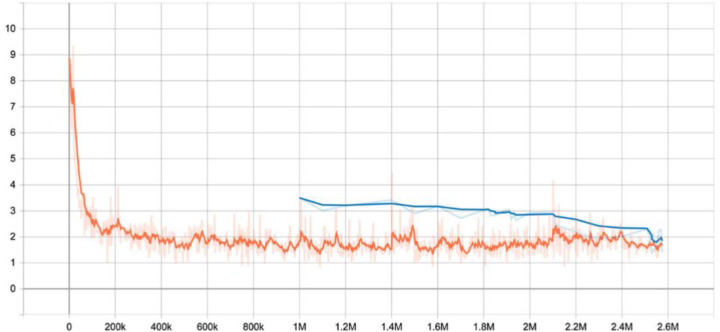
Training.

The table below summarizes the success and failure of both the covered language phrase and the forecast of the associated sentence. Figure 14 also introduces the preparatory misfortune charts.

Table 6 shows the different language modelling results. The proposed model accuracy for rouge-1, rouge-2 and rouge-3 is shown in Table 7.

**Table 6:** Language modeling results.

Training languages	Nepali perplexity
Nepali + English	140.1
Nepali	157.2
Nepali + Hindi	115.6
Nepali + English + Hindi	**109.3**

**Table 7:** Proposed model accuracy.

%	Precision	Recall	F-Measure
ROUGE-1	28.14	30.86	27.34
ROUGE-2	07.12	08.47*	07.10
ROUGE-L	28.49	25.87	25.50

## Conclusion

Text summarization software intentionally contributes a tremendous quantity of information to help readers understand the main idea of a document or article in any language. On the internet, users typically focus on the highlights of news stories, the main concept of needed information, journals, film reviews, or an overview of current scientific advances. NLP specialists focus on meeting the need for automatic summaries due to the abundance of internet information available nowadays. The outcomes of the suggested architecture demonstrate unequivocally that a serious summarizing system for Urdu texts may yield promising summaries. While keeping the idea of the source document and performing the paraphrasing to create links between the different summary sentences, the summaries generated by using the automatic abstractive text summarization architecture can compete with human-generated summaries, as is clear from the evaluation results. Additional studies in this area may result in other types of information retrieval and summaries from texts written in Urdu.

This article proposed an approach based on a deep learning model for the Urdu language and compared its performance with the two widely used methods, such as support vector machine and logistic regression. The results show that the suggested model performs better than the other two.

##  Supplemental Information

10.7717/peerj-cs.1176/supp-1 Supplemental Information 1 Source code for the project

10.7717/peerj-cs.1176/supp-2 Supplemental Information 2 Dataset from Mendeley

## Additional Information and Declarations

### Data Availability

The following information was supplied regarding data availability: The data is publicly available at Mendeley: Hussain, Khalid; Mughal, Nimra; Ali, Irfan; Hassan, Saif; Daudpota, Sher Muhammad (2021), “Urdu News Dataset 1M”, Mendeley Data, V3, doi: 10.17632/834vsxnb99.3.
